# Off‐pump versus on‐pump coronary artery bypass grafting for octogenarians: A meta‐analysis involving 146 372 patients

**DOI:** 10.1002/clc.23794

**Published:** 2022-03-10

**Authors:** Lifu Sun, Meijing Zhou, Yumeng Ji, Xufeng Wang, Xiaowei Wang

**Affiliations:** ^1^ Department of Cardiovascular Surgery The First Affiliated Hospital of Nanjing Medical University Nanjing China; ^2^ Department of Endocrine The First Affiliated Hospital of Nanjing Medical University Nanjing China

**Keywords:** atrial fibrillation, early mortality, meta‐analysis, octogenarians, off‐pump coronary artery bypass grafting, prolonged ventilation, reoperation for bleeding, stroke

## Abstract

There is an ongoing debate concerning the optimal surgical option of myocardial revascularization for octogenarians. The current meta‐analysis aimed to compare clinical outcomes following off‐pump coronary artery bypass grafting (OPCABG) or conventional coronary artery bypass grafting (CCABG) in octogenarians. PubMed, Cochrane, Web of Science, and EMBASE databases were searched to identify eligible studies from inception to March 2021. The analysis was performed using STATA 15.1. A literature search yielded 18 retrospective studies involving 146 372 patients (OPCABG = 44 522 vs. CCABG = 101 850). Pooled analysis showed a strong trend toward reducing mortality risk in the OPCABG group (odds ratio: 0.75, 95% confidence interval: 0.56–1.00, *p* = .05). However, it did not reach statistical significance. The sensitive analysis demonstrated that OPCABG was less likely to cause death than CCABG. There were comparable data in myocardial infarction, renal failure, deep sternal wound infection, and hospital stays between the two groups, although the incidence of stroke, atrial fibrillation, prolonged ventilation, and reoperation for bleeding was significantly lower in the OPCAGB group. OPCABG may be an effective surgical strategy for myocardial revascularization, especially in reducing the incidence of postoperative stroke, atrial fibrillation, prolonged ventilation, and reoperation for bleeding.

## 1 INTRODUCTION

Conventional coronary artery bypass grafting (CCABG) was the gold standard therapy for patients with complex coronary artery disease.^1^ However, this method may cause adverse effects (e.g., myocardial ischemia damage, aortic damage, coagulation problems) due to adopting cardiopulmonary bypass (CPB).^1^ Additionally, biting side clamps could result in the embolization of atherosclerotic material and sequentially bring out neurological events. Off‐pump coronary artery bypass grafting (OPCABG) was advocated subsequently for its benefits in avoiding the inherent risks linked with CPB and cardioplegic arrest. Although this approach has tangible clinical benefits, surgical techniques are more challenging. Furthermore, OPCABG has certain limitations in graft patency and revascularization integrity.^1^ The debate concerning the optimal surgical option of myocardial revascularization for octogenarians is still ongoing.

A recent editorial comment of a meta‐analysis comparing the long‐term outcomes of OPCABG versus CCABG pointed out that the discussion should be refocused from evaluating each strategy overall to investigating specifically which categories of patients will benefit more from which technique.^2^ One subset of interest in this is older adults, especially octogenarians. With life expectancy rising, an increasing number of octogenarians meet the criteria for coronary artery surgery. CCABG or OPCABG may provide adequate survival improvements to patients aged 80 years and above.^3^ However, due to age‐related comorbidities and fragility, this population is more susceptible to coronary artery surgery's adverse effects.^4^, ^5^ As a result, there is a pressing need to investigate which coronary artery bypass grafting strategy is superior for octogenarians.

Several meta‐analyses on this topic have been published.^6^, ^7^, ^8^ However, they did not resolve whether CCABG or OPCABG is better for octogenarians. For example, Pawlaczyk et al.^6^ and Khan et al.^8^ reported that the OPCABG group had reduced early death rates, while Altarabsheh et al.^7^ found comparable early mortality between the two groups. In addition, previous meta‐analyses paid less attention to the outcome of prolonged ventilation and reoperation for bleeding, whereas both these postoperative complications are well known to affect early mortality and in‐hospital medical cost.^9^, ^10^ Some large‐scale retrospective studies have recently emerged.^11^, ^12^, ^13^ Therefore, this study aimed to conduct an updated meta‐analysis to investigate whether preferentially offering OPCABG to octogenarians is more helpful in reducing mortality and other surgical outcomes (such as stroke, atrial fibrillation) than CCABG.

## 2 METHODS

### 2.1 Protocol and registration

This study was conducted following the Preferred Reporting Items for Systematic Review and Meta‐Analyses guidelines (PRISMA).^14^ The protocol of this meta‐analysis was registered on the International Prospective Register of Systematic Reviews (PROSPERO) with the registration number CRD42021249717.

### 2.2 Search strategy

We searched PubMed, Cochrane Review and CENTRAL databases, Web of Science, and EMBASE from inception to March 2021 for studies about coronary artery surgery in octogenarians. We adopted search terms as following: (a) Off‐Pump [Title/Abstract] OR On Pump [Title/Abstract] OR beating heart [Title/Abstract]; (b) Coronary revascularization [Title/Abstract] OR Coronary artery bypass graft [Title/Abstract] OR Coronary artery surgery [Title/Abstract] OR CABG [Title/Abstract]; and (c) Elderly [Title/Abstract] OR Old [Title/Abstract] OR Octogenarian [Title/Abstract]. The search terms were modified for each individual database. In addition, we performed a hand search of all included publications' reference lists to identify any eligible studies.

### 2.3 Study selection

Two investigators (L. S. and M. Z.) performed the initial screening according to the title and abstract independently. The identified studies were then followed by a full‐text screening by two independent investigators (L. S. and M. Z.). The two investigators determined final included articles according to the following prespecified criteria: (a) studies comparing clinical outcomes regarding OPCABG versus CCABG in patients older than 80 years; (b) studies could be randomized, nonrandomized, or observational; and (c) articles reporting at least endpoint concerning early mortality. Studies were excluded if they: (a) were duplicate publications; (b) did not state the interesting outcome; and (c) were published as conference abstracts, comments, letters, or editorials. Except this, if multiple articles reported the same patient cohort, we chose the latest articles or the ones with the most detailed information. Discrepancies between the two investigators were resolved by discussion with a senior investigator until reaching a consensus.

### 2.4 Outcomes of interest

The primary endpoint of interest was early mortality, including postoperative, in‐hospital, or 30‐day mortality.^7^ Secondary endpoints of interest were postoperative stroke, atrial fibrillation, renal failure, reoperation for bleeding, deep sternal wound infection, myocardial infarction, prolonged ventilation (>24, 48, or 72 h), intensive care unit (ICU) stay, and hospital stay.

### 2.5 Quality assessment

Two reviewers (L. S. and M. Z.) independently assessed the quality of each included nonrandomized study based on the modified Newcastle‐Ottawa scale (NOS).^15^ Studies were evaluated by examining three aspects: participants selection, comparability of off‐pump and on‐pump groups, and outcomes assessment. Disagreements were resolved by discussion with a senior investigator. Inter‐rater agreement was assessed using Cohen's *κ* coefficient.

### 2.6 Data collection and statistical analysis

Two investigators independently performed data extraction using a standardized form created by Excel software (Microsoft). The following information was collected: first author, publication year, country where the study was conducted, participant demographic and clinical characteristics, study design, and interesting outcomes.

All statistical analyses were performed using the Stata v15.1 software. Categorical variables were presented as frequencies, and continuous variables were reported as mean ± standard deviation. CCABG and OPCABG were regarded as control and experimental arms for each analysis. We calculated pooled odds ratios (OR) for the treatment effect of categorical variables and weighted mean differences for continuous variables, with the corresponding 95% confidence interval (CI). *p* < .05 was considered statistically significant. The meta‐analysis used the random‐effects model (inverse‐variance method) to obtain conservative pooled estimates. Heterogeneity was assessed with the *Q* and *I* ^2^ tests. The thresholds of high, moderate, and low heterogeneity were *I* ^2^ of 75% or more, 50%–74%, and 25%–49%, respectively.^16^ If moderate or high heterogeneity exists, we performed univariate meta‐regression to explore the source of inconsistency between studies. The following study characteristics were considered for heterogeneity: sample size, study quality (NOS score), the region where participants were recruited, and study design. Furthermore, sensitivity analysis was performed in two ways: (a) by combining studies that conducted propensity‐matched analysis and (b) by pooling data excluding the study with the largest sample size if the number of combined studies is >10. According to previous recommendations, 0.5 was added to the number of events to generate corrected pooled data if a specific study had 0 events in the experimental or control arm groups.^17^ Studies with 0 events in both groups would be excluded.^17^ Using funnel plot and Egger's linear regression test, we assessed publication bias for the primary outcome.^18^ Significant publication bias exists if *p *value is <.1. When there is significant publication bias, an adjusted analysis was performed using the trim‐and‐fill method to estimate the effect of potential publication bias on pooled effect size.^19^

## 3 RESULTS

### 3.1 Study selection and characteristics

**Figure 1:**
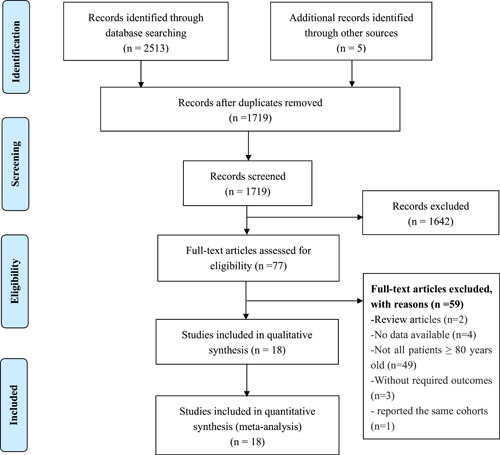
Preferred Reporting Items for Systematic Review and Meta‐Analyses guidelines (PRISMA) flowchart of the selection process

A total of 2518 articles were retrieved through database searches. After removing duplicates, 1719 records remained screened according to the title and abstract. Of those, 77 full‐text articles were further assessed for eligibility. Finally, 18 retrospective trials were included in the meta‐analysis, involving 44 522 and 101 850 participants in the OPCABG and CCABG groups, respectively.^11^, ^12^, ^13^, ^20^, ^21^, ^22^, ^23^, ^24^, ^25^, ^26^, ^27^, ^28^, ^29^, ^30^, ^31^, ^32^, ^33^, ^34^ Figure 1 illustrates the detailed review process.

The included articles were published between 2000 and 2020 and were performed in North America, Europe, and Asia. Among 18 publications, three described sample sizes were >1000.^11^, ^12^, ^13^ Propensity‐matched data were reported in five studies.^11^, ^12^, ^20^, ^21^, ^22^ The scores of a modified NOS assessment of all included studies were six or greater. Two investigators had 100% agreement equating to a Cohen's *κ* of 1. The average age of patients included was between 81.8 and 84.5 years old. The percentage of female patients ranged from 12.33% to 47.21%. Basic information regarding all included studies is presented in Table 1.

**Table 1:** Basic information of included studies

Author (year)	Country	No. of patients, O/C	Female, O/C (%)	Average age, O/C	Average No. of grafts, O/C	NOS score	Outcomes
Knapik (2020)^11^	Poland	1813/1813	35.1/35.5	82.0/82.0	NR	8	Early mortality, stroke, MI, RF, reoperation for bleeding, DSWI, prolonged ventilation
Suarez‐Pierre (2019)^12^	USA	128/128	46.1/46.1	83/83	2.26/2.96	8	Early mortality, stroke, AF, RF, prolonged ventilation
Benedetto (2017)^13^	USA	39 060/95 057	40.4/36.7	NR	NR	7	Early mortality, stroke, AF, RF
Vasques (2013)^20^	Finland	56/56	41.1/44.6	82.2/81.7	NR	8	Early mortality, stroke, AF, RF, reoperation for bleeding
Raja (2013)^21^	UK	73/73	11.0/21.2	82.3/81.9	NR	8	Early mortality, stroke, AF, RF, reoperation for bleeding, DSWI, prolonged ventilation
Saleh (2011)^22^	UK	107/107	37.2/25.7	81.8/82.1	NR	8	Early mortality, stroke, AF, MI, RF, reoperation for bleeding, DSWI
LaPar (2011)^23^	USA	404/1589	42.1/38.6	83.0/82.5	2.2/3.4	7	Early mortality, stroke, AF, MI, RF, prolonged ventilation
Sarin (2011)^24^	USA	540/397	45.9/44.6	82.9/82.3	2.62/2.7	7	Early mortality, stroke, AF, MI, RF, reoperation for bleeding, DSWI
Serrão (2010)^25^	Portugal	65/36	36.9/33.3	82.7/82.2	NR	7	Early mortality, stroke
Tugtekin (2007)^26^	Germany	107/237	36.4/36.3	82/82	NR	7	Early mortality, stroke, AF, MI, RF, reoperation for bleeding, prolonged ventilation
Nagpal (2006)^27^	UK	131/105	42.8/26.7	82.1/82.3	2.76/3.16	7	Early mortality, stroke, AF, RF, MI, reoperation for bleeding, prolonged ventilation, DSWI
D'Alfonso (2004)^28^	Italy	73/41	32.9/41.5	81.8/82.1	NR	7	Early mortality, AF, reoperation for bleeding
Lin (2003)^29^	China	17/12	23.5/33.3	82.2/83.5	3.1/3.0	6	Early mortality, stroke, AF, MI, RF, reoperation for bleeding, DSWI
Shimokawa (2003)^30^	Japan	25/18	40.0/44.4	82.2/81.9	2.0/2.8	6	Early mortality, AF, reoperation for bleeding, DSWI
Demaria (2002)^31^	Canada	62/63	NR	NR	2.6/2.9	8	Early mortality, stroke, AF, MI, RF, reoperation for bleeding, prolonged ventilation
Hoff (2002)^32^	USA	59/169	48.3/45.6	84.2/84.1	2.9/3.5	7	Early mortality, stroke, AF, RF, reoperation for bleeding, prolonged ventilation, DSWI
Yokoyama (2002)^33^	USA	28/58	NR	NR	3.2/4.1	6	Early mortality, RF, reoperation for bleeding, prolonged ventilation
Ricci (2000)^34^	USA	97/172	49.5/45.9	84.5/83.6	NR	6	Early mortality, stroke, MI, RF, prolonged ventilation, DSWI

Abbreviations: AF, atrial fibrillation; C, CCABG; DSWI, deep sternal wound infection; MI, myocardial infarction; NOS, Newcastle‐Ottawa scale; NR, not report; O, OPCABG; RF, renal failure.

Nine studies reported the number of grafts between the two groups. Three studies showed that more internal thoracic arteries were used in the OPCABG cohort than in the CCABG cohort. Five studies described similar left ventricular ejection fractions beween groups. Table 2 demonstrates the characteristics and preoperative comorbidities of patients.

**Table 2:** Characteristics and preoperative comorbidities of patients

Author (year), O/C	Hypertension, O/C (%)	DM, O/C (%)	CVA, O/C (%)	PVD, O/C (%)	COPD, O/C (%)	RD, O/C (%)	MI, O/C (%)	ITA use, O/C (%)	Urgent surgery, O/C (%)	Mean LVEF, O/C (%)
Knapik (2020)^11^	90.0/90.0	34.2/32	3.9/4.0	11.6/11.1	6.8/6.6	13.4/13.8	28.3/33.1	NR	45.6/44.3	NR
Suarez‐Pierre (2019)^12^	93.0/93.0	35.9/34.4	13.3/5.5	21.9/18.8	NR	NR	60.9/68.8	97.7/93.0	78.1/81.3	NR
Benedetto (2017)^13^	70.9/72.3	23.6/25	NR	18.8/17.1	21.9/19.4	15.6/15.2	NR	NR	59.5/60.5	NR
Vasques (2013)^20^	55.4/57.1	16.1/17.9	1.8/5.4	5.4/0	NR	NR	53.6/48.2	NR	7.1/3.6	NR
Raja (2013)^21^	78.1/74	20.5/16.4	2.7/4.1	8.2/6.9	5.5/4.2	6.8/4.1	15.1/13.9	NR	23.3/19.2	NR
Saleh (2011)^22^	68.6/65.2	21.2/19.3	7.7/11.8	13.5/16.0	NR	8.3/13.4	59.6/56.2	NR	37.2/42.3	NR
LaPar (2011)^23^	81.2/83.9	27.2/30.5	11.6/8.3	19.6/18.9	NR	7.4/5.8	NR	91.9/82.3	59.7/61.7	NR
Sarin (2011)^24^	86.3/77.3	28.0/27.2	11.5/13.9	15.6/6.1	11.5/2.5	9.1/8.1	49.8/50.1	NR	25.7/19.1	52.1/50.6
Serrão (2010)^25^	73.8/75.0	26.2/27.8	7.8/8.3	20.0/30.6	NR	NR	NR	NR	30.7/27.8	NR
Tugtekin (2007)^26^	67.2/60.5	31.8/46.2	9.8/5.0	9.3/7.6	9.8/6.7	30.3/25.6	46.7/59.1	NR	NR	55/56.2
Nagpal (2006)^27^	77.1/58.1	19.9/15.2	10.7/7.6	15.3/16.2	12.2/12.4	31.3/23.8	47.3/38.1	NR	18.4/17.1	NR
D'Alfonso (2004)^28^	56.2/41.5	26.0/24.0	9.6/9.8	5.5/4.9	9.6/2.4	NR	NR	NR	NR	47/47
Lin (2003)^29^	58.8/58.3	38.9/25.0	5.9/25.0	NR	17.6/8.3	5.9/8.3	23.5/25.0	NR	NR	53.4/42.0
Shimokawa (2003)^30^	88.0/83.3	32.0/22.2	60.0/50.0	24.0/27.8	24.0/16.7	4.0/0	NR	NR	32.0/55.6	NR
Demaria (2002)^31^	NR	NR	NR	NR	NR	NR	21.0/22.2	NR	NR	NR
Hoff (2002)^32^	NR	30.0/23.1	10.0/8.3	NR	18.3/11.8	0/0	27.4/24.2	85/52.7	0/0	NR
Yokoyama (2002)^33^	NR	NR	NR	NR	NR	NR	NR	NR	NR	NR
Ricci (2000)^34^	83.5/80.8	15.5/19.8	17.5/14.0	7.2/17.4	32.0/27.9	2.1/1.7	62.9/62.2	NR	54.6/62.2	50/50

Abbreviations: C, CCABG; COPD, chronic obstructive pulmonary disease; CVA, cerebrovascular accident; DM, diabetes mellitus; ITA, internal thoracic artery; LVEF, left ventricular ejection fractions; MI, myocardial infarction; NR, not report; O, OPCABG; PVD, peripheral vascular disease; RD, renal disease.

### 3.2 Primary outcomes

**Figure 2:**
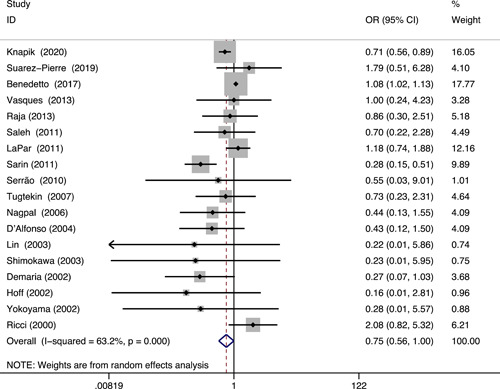
Forest plot of comparison with the outcome of early mortality. CI, confidence interval; OR, odds ratio

All 18 studies reported early mortality.^11^, ^12^, ^13^, ^20^, ^21^, ^22^, ^23^, ^24^, ^25^, ^26^, ^27^, ^28^, ^29^, ^30^, ^31^, ^32^, ^33^, ^34^ The death occurred in 2356 of 42 845 patients in the OPCABG group, and in the CCAB group, it occurred in 5257 of 100 131. Figure 2 displays the forest plots for early mortality. A strong trend toward reducing mortality risk was observed in the OPCABG group. However, it was not statistically significant (pooled OR: 0.75, 95% CI: 0.56–1.00, *p* = .05). Moderate heterogeneity across studies existed (*I* ^2^ = 63.2%, *p* < .001). In the random‐effects univariate meta‐regression model, we included the following covariates individually: study quality, sample size, study design, and region (North America, Europe, Asia). No variable can explain the heterogeneity, suggesting clinical inconsistency between studies. A list of overall coefficients is provided in Table S1. Sensitivity analysis was performed in two ways: (a) by pooling data excluding a study by Benedetto et al.^13^ and (b) by only combining studies with propensity‐matched design.^11^, ^12^, ^20^, ^21^, ^22^ The results of sensitivity analysis suggested that OPCABG was less likely to cause death than CCABG (*p* < .05; Table 3).

**Table 3:** Summary of results of the meta‐analysis

Outcome	Included studies	No. of patients, O/C	Pooled OR/SMD (95% CI)	*I* ^2^ and *p *value	Sensitivity analysis performed	Adjusted OR (95% CI)
*Primary outcome*						
Early mortality	18	42 845/100 131	0.75 (0.56, 1.00)	*I* ^2^ = 63.2%, *p* < .001	(1) Excluding Benedetto et al.	0.69 (0.50, 0.95)
					(2) Pooling propensity‐matched studies	0.74 (0.59, 0.92)
*Secondary outcomes*						
Stroke	15	42 719/100 014	0.70 (0.61, 0.80)	*I* ^2^ = 2.1%, *p* = .43		
Myocardial infarction		3278/4495	0.92 (0.53, 1.59)	*I* ^2^ = 26.3%, *p* = .21		
Renal failure	14	42 620/99 973	1.04 (0.97, 1.12)	*I* ^2^ = 0.0%, *p* = .84		
Atrial fibrillation	14	40 842/98 052	0.89 (0.87, 0.91)	*I* ^2^ = 0.0%, *p* = .62		
Prolonged ventilation	10	2902/4407	0.69 (0.58, 0.81)	*I* ^2^ = *0.0%, p* = *0.64*		
Reoperation for bleeding	13	3091/3149	0.77 (0.61, 0.97)	*I* ^2^ = 0.0%, *p* = .75		
Deep sternal wound infection	9	2862/2866	1.02 (0.66, 1.57)	*I* ^2^ = 0.0%, *p* = .66		
ICU stays	11	1650/2785	−0.21 (‐0.39, −0.03)	*I* ^2^ = 81.4%, *p* < .001	Pooling propensity‐matched studies	−0.21 (−0.49, 0.07)
Hospital stays	12	42 356/99 439	−0.06 (−0.15, 0.02)	*I* ^2^ = 73.5%, *p* < .001	(1) Excluding Benedetto et al.	−0.13 (−0.25, 0.00)
					(2) Pooling propensity‐matched studies	−0.00 (−0.07, 0.06)

Abbreviations: ICU, intensive care unit; OR, odds ratio; SMD, standard mean difference.

It is well known that “positive” studies are more likely to be reported in peer‐reviewed publications.^6^ In this meta‐analysis, we adopted two widely used methods, the funnel plot and Egger's linear regression test, to calculate publication bias. The funnel plot (Figure S1) shows the asymmetrical distribution of studies, suggesting that publication bias exists among studies.^18^ The result of Egger's test also confirmed statistically significant publication bias (*p* = .019). Nonetheless, the result of the trim‐and‐fill test indicated the publication bias had no effect on the pooled estimate of the primary endpoint (no trimming performed and the pooled effect size unchanged).

### 3.3 Secondary outcomes

#### 3.3.1 Stroke

Postoperative stroke was defined as a new neurological deficit lasting over 24 h.^6^ Fifteen studies provided information about postoperative stroke, involving 142 733 participants (OPCABG vs. CCABG: 42 719 vs. 100 014).^11^, ^12^, ^13^, ^20^, ^21^, ^22^, ^23^, ^24^, ^25^, ^26^, ^27^, ^29^, ^31^, ^32^, ^34^ The incidence of postoperative stroke was 1.8% and 2.5% in the OPCABG and CCABG groups, respectively. The pooled results suggested a significant decrease in postoperative stroke risk in the OPCABG cohort than in the CCABG cohort (pooled OR: 0.70, 95% CI: 0.61–0.80, *p* < .001; *I* ^2^ = 2.1%, *p* = .43; Table 3). No statistical heterogeneity between studies was observed.

#### 3.3.2 Prolonged ventilation

Prolonged ventilation (>24, 48, or 72 h) was observed in ten studies.^11^, ^12^, ^21^, ^23^, ^26^, ^27^, ^31^, ^32^, ^33^, ^34^ The total number of participants receiving OPCABG was 2902, and 4407 patients underwent CCABG. The pooled data demonstrated higher prolonged ventilation risk in the CCABG group than in the OPCABG group (12.5% vs. 8.3%, pooled OR: 0.69, 95% CI: 0.58–0.81, *p* < .001). No statistical inconsistency between studies was observed (*I* ^2^ = 0.0%, *p* = .64; Table 3).

#### 3.3.3 Reoperation for bleeding

Thirteen studies with 3091 patients in the OPCABG group and 3149 in the CCABG group provided the data of reoperation for bleeding.^11^, ^20^, ^21^, ^22^, ^24^, ^26^, ^27^, ^28^, ^29^, ^30^, ^31^, ^32^, ^33^ The trial by Lin et al.^29^ and Shimokawa et al.^30^ had to be excluded owing to unable to calculate OR because of 0 events in the two groups. Hence, we combined ten studies, and the result showed that OPCABG was associated with less reoperation for bleeding than CCABG (4.4 vs. 5.6%, pooled OR: 0.77, 95% CI: 0.61–0.97, *p* = .03; *I* ^2^ = 0.0%, *p* = .75; Table 3).

#### 3.3.4 Myocardial infarction

Nine studies (OPCABG vs. CCABG: 3278 vs. 4495 patients) compared the incidence of postoperative myocardial infarction of the two surgery strategies.^11^, ^22^, ^23^, ^24^, ^26^, ^27^, ^29^, ^31^, ^34^ A total of 42 and 55 patients in the OPCABG and CCAB cohorts reported myocardial infarctions, respectively. Pooled analysis demonstrated no significant difference between the two groups (pooled OR: 0.92, 95% CI: 0.53–1.59, *p* = .77; Table 3). There was low heterogeneity across studies (*I* ^2^ = 26.3%, *p* = .21).

#### 3.3.5 Renal failure

We defined renal failure as a new need for any form of renal replacement therapy postoperatively, such as hemodialysis and hemofiltration. The incidence of renal failure between the two groups was reported in 14 trials.^11^, ^12^, ^13^, ^20^, ^21^, ^22^, ^23^, ^26^, ^27^, ^29^, ^32^, ^33^, ^34^ The rate of postoperative renal failure was 2.7% in the OPCABG and 2.5% in the CCABG group. The combined result showed that the occurrence of postoperative renal failure was comparable in the two groups (pooled OR: 1.04, 95% CI: 0.97–1.12, *p* = .25; *I* ^2^ = 0.0%, *p* = .84; Table 3).

#### 3.3.6 Atrial fibrillation

Fourteen trials reported postoperative atrial fibrillation (OPCABG vs. CCABG: 40 842 vs. 98 052 patients).^12^, ^13^, ^20^, ^21^, ^22^, ^23^, ^24^, ^26^, ^27^, ^28^, ^29^, ^30^, ^31^, ^32^ This event arose in 42.6% and 45.5% of patients in the OPCABG and CCABG cohorts, respectively. The incidence of postoperative atrial fibrillation was significantly lower in the OPCAGB group than in the CCABG group (pooled OR: 0.89, 95% CI: 0.87–0.91, *p* < .001; *I* ^2^ = 0.0%, *p* = .62; Table 3).

#### 3.3.7 Deep sternal wound infection

Deep sternal wound infection was reported in nine studies, including 2862 and 2866 patients in OPCABG and CCABG cohorts, respectively.^11^, ^21^, ^22^, ^24^, ^27^, ^29^, ^30^, ^32^, ^34^ We had to eliminate studies with 0 events in both groups for calculating OR.^22^, ^29^, ^30^, ^32^ Therefore, data from five studies was pooled,^11^, ^21^, ^24^, ^27^, ^34^ and the result displayed no statistically significant difference in the risk of deep sternal wound infection concerning the two surgery strategies (1.5 vs. 1.4%, pooled OR: 1.02, 95% CI: 0.66–1.57, *p* = .94; *I* ^2^ = 0.0%, *p* = .66; Table 3).

#### 3.3.8 ICU stays

Eleven studies provided data concerning ICU stays, comprising 4435 patients.^12^, ^20^, ^21^, ^22^, ^23^, ^24^, ^26^, ^27^, ^29^, ^30^, ^31^ The pooled analysis revealed that ICU stays were significantly shorter in patients who underwent OPCABG than those who received CCABG (pooled standard mean difference [SMD]: −0.21, 95% CI: −0.39 to −0.03, *p* = .02; Table 3). High heterogeneity was detected between 11 studies (*I* ^2^ = 81.4%, *p* < .001). The random‐effects meta‐regression model was conducted to explore the source of inconsistency. However, only “region” was found to contribute significantly to heterogeneity. A list of overall coefficients was provided in Table 2. The sensitivity analysis result by only combining propensity‐matched studies showed no statistical difference in ICU stays between the two groups (pooled SMD: −0.21, 95% CI: −0.49 to 0.07, *p* = .14; Table 3).^12^, ^20^, ^21^, ^22^

#### 3.3.9 Hospital stays

Twelve studies with 141 795 patients reported hospital stays.^11^, ^12^, ^21^, ^22^, ^23^, ^24^, ^25^, ^27^, ^29^, ^30^, ^31^, ^32^ The pooled result demonstrated no significant difference in hospital stays no matter what kind of surgeries patients followed (pooled SMD: −0.06, 95% CI: −0.15 to 0.02, *p* = .14; *I* ^2^ = 73.5%, *p* < .001). In the random‐effects meta‐regression model, only “region” could partially interpret heterogeneity across these studies. Table S3 presented a list of all coefficients. Sensitivity analysis by only pooling studies with propensity‐matched design^11^, ^12^, ^21^, ^22^ and excluding Benedetto et al.^13^ also affirmed our original result (Table 3).

### 3.4 The overall quality of evidence

All outcomes' overall quality of evidence was downgraded to “very low,” mainly due to limited propensity‐matched data, moderate heterogeneity, or the inconsistent pooled data before and after the sensitivity analysis. Results from the GRADEpro analysis incorporating the assessment of evidence quality for outcomes are presented in Table S4.

## 4 DISCUSSION

This systematic review aimed to investigate the superiority of OPCABG and CCABG in octogenarians. Of 18 studies included, all were observational studies and were further assessed using meta‐analysis.

Before conducting the sensitivity analysis, the current study found a strong tendency to lower early death incidence in the OPCABG group, although it did not reach statistical significance. Following the sensitivity analysis, pooled data revealed that the CCABG group had significantly greater mortality than the OPCABG group. Altarabsheh et al.^7^ observed no difference in early mortality among octogenarians receiving CCABG or OPCABG in a prior meta‐analysis. Another two meta‐analyses, however, reported that octogenarians in the OPCABG group had a reduced in‐hospital death rate.^8^, ^9^ The discrepancy in these conclusions may be due to differences in endpoint definitions. Mauldon et al.^35^ conducted a meta‐analysis to investigate the effect of age on outcomes after OPCABG or CCABG. They found that the patient's age had no bearing on the chosen surgical method in the short‐term (<30 days) mortality. It was worth highlighting that the average age of the patients enrolled in their study ranged from 51.5 to 78.4 years old, while the age of patients recruited in our study is above 80 years old. Thus, the finding reported by Mauldon et al. had significant limits. Owing to the contradictory pooled estimate before and after sensitivity analysis, more well‐designed studies with larger samples were needed to detect the effects of OPCABG in reducing mortality in octogenarians because of weak evidence and heterogeneity.

The pooled results indicated that OPCABG could be more effective in reducing postoperative stroke rates. This result followed previous meta‐analyses' findings.^6^, ^7^, ^8^ Although multiple factors play critical roles in the pathophysiology of brain damage, there is increasing evidence that numerous microemboli generated by the heart cavity, ascending aorta, or bypass circuit could cause diffuse ischemic brain injury.^36^ A network meta‐analysis illustrated that avoiding CPB and aortic manipulation could lower the incidence of postoperative stroke, particularly in patients with a greater risk of stroke.^37^ Eliminating aortic manipulation and CPB may reduce cerebral adverse events by preventing intraoperative atherosclerotic arterial embolism from entering the circulation.^38^ None of the trials included in this study reported an aortic operation in the OPCABG cohort. Several studies included in this meta‐analysis were observed placing a side clamp on the aorta. Therefore, we assumed that the lower stroke rate in the OPCABG group might be mainly ascribed to the avoidance of aortic cannulation and cross‐clamping.

The pooled results showed that the CCABG group might have a substantially higher risk of prolonged ventilation. A recent randomized trial displayed that patients receiving CCABG had double the ventilation time as those undergoing OPCABG.^10^ Prolonged ventilation is affected by multiple factors, involving age, left ventricular dysfunction, peripheral vascular disease, chronic obstructive pulmonary disease (a critical, independent predicting factor for prolonged ventilation), and intraoperative factors.^39^ Considering that there is no statistical difference in most demographic and social characteristics between the two groups, a systemic inflammatory response with a cascade of cytokines produced by CPB might be an essential explanation for prolonged ventilation in the CCABG group in our study. Additionally, Chiarenza et al.^40^ also mentioned that postoperative stroke and reoperation for bleeding were independent risk factors for prolonged ventilation, while our study suggested that the incidence of postoperative stroke and reoperation for bleeding was higher in the CCABG group. Hence, finding a decreased prolonged ventilation rate in the OPCABG group could be plausible.

Interestingly, the pooled estimate revealed that the incidence of reoperation for bleeding might be lower in the OPCABG group, inconsistent with Khan and his colleagues' conclusions.^8^ The discrepancy could be caused by different sample sizes between studies since the power to detect statistical significance increases with growing sample size, especially when the low events rate. The potential interpretation of this finding might be postoperative coagulation disorders resulting from the inflammatory response (such as complement and leukocytes activation, proinflammatory cytokine release, and increased production of oxygen‐free radicals) after CPB, as well as minimal dissection.^41^

Moreover, this study found a decreased incidence of atrial fibrillation in patients receiving OPCABG, which contradicted prior meta‐analyses' findings.^6^, ^7^, ^8^ Previous meta‐analyses' negative results may be due to a small sample size that is not powerful enough to identify differences between groups. Patients in the OPCABG group refrain from atrial cannulation could be one primary reason for reduced atrial fibrillation in the OPCABG group. Furthermore, postoperative lower incidence of atrial fibrillation can predict a reduced postoperative stroke rate independently.^42^

Of note, neither this study nor earlier meta‐analyses identified the optimal method of myocardial revascularization (CCABG or OPCABG) for octogenarians in decreasing postoperative myocardial infarction, renal failure, and deep sternal wound infection. Furthermore, contrary to former meta‐analyses, this study found no significant difference in hospital stays between the two groups. As we know, apart from surgical factors, various factors influence hospital stays, such as the patients' prior physical status and personal preferences.

In the GRADE system, observational studies start with a “low quality” rating.^43^ Any potential influencing factors (including study limitations, inconsistent results, indirectness of evidence, imprecision of results, or publication bias) will cause the quality of observational studies to be downgraded.^43^ In this study, the "very low" quality of evidence for most outcomes is mainly due to limitations caused by the study design. Although the included partial studies did not adopt propensity‐matched designs, the sample sizes of these studies were considerable, increasing the credibility of the pooled estimate to a certain extent. Additionally, given the lack of relevant randomized controlled trials (RCTs), we may regard available observational studies as reliable evidence on this topic. Further well‐designed RCTs regarding this issue will be required to verify our conclusions in the future.

### 4.1 Limitations

The present meta‐analysis has several restraints, and the results should be interpreted with caution. First, all the papers included were retrospective studies, which may lead to selection bias. Second, clinical heterogeneity exists across studies to some extent. As we know, surgeons' experience and the volume of performed procedures can impact the endpoints of CABG.^6^ Nevertheless, none of the studies included in our meta‐analysis reported data about the volume of performed procedures in medical institutions or how familiar surgeons were with the surgery techniques, which possibly increased clinical heterogeneity and affected the pooled findings. To correct this limitation, we used a random‐effects model to integrate the data. More importantly, we encourage upcoming studies on this topic to consider presenting detailed data about surgeons' experience and the volume of performed procedures to facilitate the further use of meta‐analysis to compare which strategy of CABG is better. Third, there was a significant publication bias in the study, but the result of the trim‐and‐fill revealed no impact of publication bias on pooled estimates. Additionally, this study could not provide convincing conclusions regarding the superiority of these two strategies in reducing postoperative mortality and ICU stays in octogenarians owing to weak evidence and heterogeneity. Furthermore, although graft patency rate is an essential indicator reflecting the effect of CABG, we know little about graft patency rate during follow‐up in our included studies. Only Shimokawa et al. reported a comparable graft patency rate after OPCABG and CCABG. There is evidence suggesting better outcomes with the use of dual antiplatelet therapy following CABG.^44^ However, no available data supported this viewpoint in our included studies due to the lack of long‐term follow‐up. Thus, we recommend that future studies conduct long‐term follow‐up and provide more information concerning the use of dual antiplatelet therapy and graft patency rate to compare the long‐term outcomes of OPCABG and CCABG.

## 5 CONCLUSIONS

OPCABG may be an attractive myocardial revascularization strategy with reduced incidence of early death, stroke, atrial fibrillation, prolonged ventilation, and reoperation for bleeding compared with CCABG for octogenarians. Preferentially offering OPCABG to this subset seems helpful in decreasing the economic burden on the patients and healthcare providers, considering the lower rate of prolonged ventilation and reoperation for bleeding.

## CONFLICT OF INTERESTS

The authors declare that there are no conflict of interests.

## AUTHOR CONTRIBUTIONS

Conceptualization: Lifu Sun and Meijing Zhou. Methodology: Lifu Sun and Meijing Zhou. Formal analysis: Lifu Sun, Yumeng Ji and Xufeng Wang. Investigation: Lifu Sun and Meijing Zhou. Writing—original draft preparation: Lifu Sun and Meijing Zhou. Writing—review and editing: Xiaowei Wang. Visualization: Lifu Sun. All authors have read and agreed to the manuscript submitted.

## Supporting information

Funnel plot for early mortality.

Supporting information.

Supporting information.

## Data Availability

The data used to support the findings of this study are available from the corresponding author upon request.
