# Supplementary material for: Off‐pump versus on‐pump coronary artery bypass grafting for octogenarians: A meta‐analysis involving 146 372 patients
Source: Clin Cardiol. 2022 Mar 10;45(4):331–41. doi: 10.1002/clc.23794 (PMC9019872; doi:10.1002/clc.23794)
Supplement: Supplementary file 1 — Funnel plot for early mortality. [file CLC-45-331-s002.pdf]

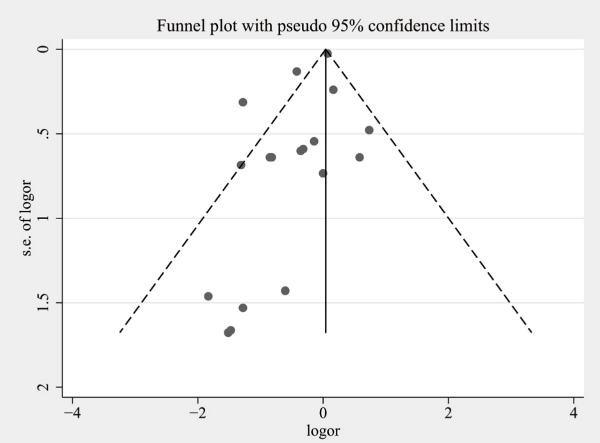

Supplementary Figure 1. Funnel plot for early mortality
