# Supplementary material for: Off‐pump versus on‐pump coronary artery bypass grafting for octogenarians: A meta‐analysis involving 146 372 patients
Source: Clin Cardiol. 2022 Mar 10;45(4):331–41. doi: 10.1002/clc.23794 (PMC9019872; doi:10.1002/clc.23794)
Supplement: Supplementary file 2 — Supporting information. [file CLC-45-331-s003.docx]

**Supplementary table 1.** Meta-regression coefficient for early mortality

|  | **Estimate** | **SE** | ***t*** | ***P*** | **Lower CI** | **Upper CI** |
| --- | --- | --- | --- | --- | --- | --- |
| Quality | -0.07 | 0.27 | -0.24 | 0.813 | -0.65 | 0.51 |
| Sample | <0.001 | <0.001 | 0.97 | 0.346 | <-0.001 | <0.001 |
| _Idesign_1 | -0.27 | 0.38 | -0.71 | 0.489 | -1.09 | 0.543 |
| _Iregion_1 | 0.21 | 0.35 | 0.61 | 0.549 | -0.53 | 0.95 |
| _Iregion_3 | -1.07 | 1.25 | -0.86 | 0.403 | -3.74 | 1.59 |

Note: CI, confidence interval; SE, standard error; _Idesign_1, design without propensity-matched analysis;

_Iregion_1, North America; _Iregion_3, Asia

**Supplementary table 2.** Meta-regression coefficient for ICU stays

|  | **Estimate** | **SE** | ***t*** | ***P*** | **Lower CI** | **Upper CI** |
| --- | --- | --- | --- | --- | --- | --- |
| Quality | 0.37 | 0.21 | 1.77 | 0.110 | -1.10 | 0.85 |
| Sample | <0.001 | <0.001 | 0.45 | 0.66 | <-0.001 | <0.001 |
| _Idesign_1 | -0.10 | 0.35 | -0.28 | 0.787 | -0.89 | 0.70 |
| _Iregion_1 | 0.12 | 0.20 | 0.62 | 0.553 | -0.34 | 0.59 |
| _Iregion_3 | -1.11 | 0.39 | -2.86 | **0.021** | -2.01 | -0.21 |

Note: CI, confidence interval; SE, standard error; _Idesign_1, design without propensity-matched analysis;

_Iregion_1, North America; _Iregion_3, Asia

**Supplementary table 3.** Meta-regression coefficient for hospital stays

|  | **Estimate** | **SE** | **t** | **p** | **Lower CI** | **Upper CI** |
| --- | --- | --- | --- | --- | --- | --- |
| Quality | 0.36 | 0.17 | 2.08 | 0.065 | -0.03 | 0.74 |
| Sample | <0.001 | <0.001 | 0.58 | 0.573 | <-0.001 | <0.001 |
| _Idesign_1 | -0.18 | 0.27 | -0.67 | 0.517 | -0.77 | 0.41 |
| _Iregion_1 | -0.04 | 0.04 | -1.10 | 0.301 | -0.13 | 0.04 |
| _Iregion_3 | -1.37 | 0.33 | -4.10 | **0.003** | -2.13 | -0.61 |

CI, confidence interval; SE, standard error; _Idesign_1, design without propensity-matched analysis;

_Iregion_1, North America; _Iregion_3, Asia

| **Supplementary table 4. GRADE analysis: overall quality assessment and summary of findings** | | | | | | |
| --- | --- | --- | --- | --- | --- | --- |
| **OPCABG compared to CCABG for octogenarians** | | | | | | |
| **Patient or population**: octogenarians  **Intervention**: OPCABG  **Comparison**: CCABG | | | | | | |
| Outcomes | **Anticipated absolute effects^*^** (95% CI) | | Relative effect (95% CI) | № of participants  (studies) | Certainty of the evidence (GRADE) | Comments |
|  | **Risk with CCABG** | **Risk with OPCABG** |  |  |  |  |
| early mortality | 53 per 1,000 | **40 per 1,000** (30 to 53) | **OR 0.75** (0.56 to 1.00) | 7613 cases 135363 controls (18 observational studies) | ⨁◯◯◯ VERY LOW ^a,b,c^ |  |
| stroke | 25 per 1,000 | **17 per 1,000** (15 to 20) | **OR 0.70** (0.61 to 0.80) | 3211 cases 139522 controls (15 observational studies) | ⨁◯◯◯ VERY LOW ^a^ |  |
| renal failure | 25 per 1,000 | **26 per 1,000** (24 to 28) | **OR 1.04** (0.97 to 1.12) | 3650 cases 138943 controls (14 observational studies) | ⨁◯◯◯ VERY LOW ^a^ |  |
| atrial fibrillation | 455 per 1,000 | **426 per 1,000** (421 to 432) | **OR 0.89** (0.87 to 0.91) | 61988 cases 76906 controls (14 observational studies) | ⨁◯◯◯ VERY LOW ^d^ |  |
| Prolonged ventilation | 125 per 1,000 | **90 per 1,000** (77 to 104) | **OR 0.69** (0.58 to 0.81) | 795 cases 6514 controls (10 observational studies) | ⨁◯◯◯ VERY LOW ^e^ |  |
| Reoperation for bleeding | 56 per 1,000 | **44 per 1,000** (35 to 55) | **OR 0.77** (0.61 to 0.97) | 313 cases 5927 controls (13 observational studies) | ⨁◯◯◯ VERY LOW ^d^ |  |
| Deep sternal wound infection | 14 per 1,000 | **15 per 1,000** (9 to 22) | **OR 1.02** (0.66 to 1.57) | 85 cases 5643 controls (9 observational studies) | ⨁◯◯◯ VERY LOW ^e^ |  |
| ICU stays | — | — | **SMD -0.21** (-0.39 to -0.03) | (11 observational studies) | ⨁◯◯◯ VERY LOW ^c,e,f^ |  |
| hospital stays | — | — | **SMD -0.06** (-0.05 to 0.02) | (12 observational studies) | ⨁◯◯◯ VERY LOW ^b,e^ |  |
| ***The risk in the intervention group** (and its 95% confidence interval) is based on the assumed risk in the comparison group and the **relative effect** of the intervention (and its 95% CI).  **CI:** Confidence interval; **OR:** Odds ratio, SMD: standard mean difference | | | | | | |
| **GRADE Working Group grades of evidence** **High certainty:** We are very confident that the true effect lies close to that of the estimate of the effect **Moderate certainty:** We are moderately confident in the effect estimate: The true effect is likely to be close to the estimate of the effect, but there is a possibility that it is substantially different **Low certainty:** Our confidence in the effect estimate is limited: The true effect may be substantially different from the estimate of the effect **Very low certainty:** We have very little confidence in the effect estimate: The true effect is likely to be substantially different from the estimate of effect | | | | | | |

**Explanations**

a. Propensity-matched data was reported in five studies.

b. Moderate heterogeneity across studies existed.

c. The pooled data has changed after the sensitivity analysis performed.

d. Propensity-matched data was reported in four studies.

e. Propensity-matched data was reported in three studies.

f. High heterogeneity across studies existed.
